# Closing the Gap Between Entrustment and Resuscitation

**DOI:** 10.5811/westjem.2018.5.38176

**Published:** 2018-06-11

**Authors:** Teresa Camp-Rogers, Douglas Franzen

**Affiliations:** * William Carey University College of Osteopathic Medicine, Department of Clinical Sciences, Hattiesburg, Mississippi; † University of Washington at Harborview, Department of Emergency Medicine, Seattle, Washington

In 2014, the American Association of Medical Colleges (AAMC) and the American Association of Colleges of Osteopathic Medicine more specifically defined the skills required of graduating medical students. These skillsets are rooted in the United States’ and Canada’s movement toward a competency-based undergraduate medical education (UME) and are termed the Core “entrustable professional activities” (EPAs).^1^ EPA 10 is most germane to emergency physicians, asking that newly minted medical students be able to “recognize a patient requiring urgent or emergent care and initiate evaluation and management.^1^” EPA 10 highlights a desperate gap in UME, as evidence confirms that interns are wholly unprepared to identify and manage emergencies independently on day one of residency.^2^,^3^ Clear discrepancies remain between UME and graduate medical education (GME) that make the transition to residency challenging for novice physicians, and potentially unsafe for patients. As currently written, EPA 10 lacks depth in content and provides only a limited discussion of the importance of rapid decision-making. A clear gap exists between the educational objectives of EPA 10 and successful resuscitation practices. We advocate for restructuring and reconsideration of the curricular recommendations of EPA 10. We propose that EPA 10 include a consensus-based list of emergencies in addition to recommendations on teaching medical students situational leadership, crisis resource management, and decision-making in emergencies.

EPA 10 provides a list of emergencies to consider for an UME curriculum.^1^ While the authors attest that this list is incomplete, such deficiencies should be quickly resolved. Trauma-related injuries, cardiac arrest and the acute abdomen are absent from the list. A comprehensive, consensus-based list of emergencies should be determined by experts in the field. The list of emergencies recommended by EPA 10 should be paired with an explicit discussion of the broader skills and heuristics needed to manage common emergent problems.

In the Core EPA document, a clinical vignette is provided to paint a picture of the ideal young physician in training who successfully manages a critically ill patient.^1^ The post-graduate year 1 resident described in EPA 10 can recognize an emergency, communicate effectively with team members in emergencies, activate and carry out a plan, and accept and incorporate feedback when managing similar situations in the future. In an ideal situation, an intern does call the senior and muster the team. But how and when does one decide to activate this intended plan? Is knowledge of a disease-specific treatment algorithm (i.e., cardiac arrest, stroke protocol, etc.) both necessary and sufficient to initiate and lead the care of a critically ill patient? Two studies have demonstrated methods of assessing EPA 10 and maintain the importance of leadership in acute care situations.^4^,^5^ Leadership is mentioned in two of the AAMC General Physician Competencies, specifically Interpersonal and Communication Skills (ICS 3) and Personal and Professional Development (PPD 6).^6^ However, in the Core EPA document describing EPA 10, leadership is not explicitly mentioned.

If we describe a more realistic version of a long shift, full of complex, rapid decision-making that occurs on little sleep with limited time, then we can cultivate a curricular strategy to focus on the skills to succeed.^7^ Instead of disease-specific knowledge, the curricular approach to teach EPA 10 should foster the growth of skills such as situational leadership, crisis resource management, and recognition primed decision-making. In his book, *Decisions of Power*, Gary Klein describes the art and science of rapid decision-making.^8^

In summary, EPA 10 appropriately contends that education in resuscitation should extend to UME, and the AAMC should be applauded for pushing UME in this direction. Emergency care and resuscitation specialists are responsible for leading the path of knowledge translation for this set of skills. Educational objectives for teaching the evaluation and management of emergencies to medical students should include situational leadership, crisis resource management, and decision-making in emergencies. In the interest of patient safety and physician wellbeing, the gap between what is taught to undergraduate medical students on emergency care and what is expected on day one of residency should be the first subject that is examined. The heuristics of leadership and decision-making in emergencies has been delivered to bystanders, paramedics, and residents. Why not to medical students? It’s time to close the gap.
